# Fenofibrate Attenuated Glucose-Induced Mesangial Cells Proliferation and Extracellular Matrix Synthesis via PI3K/AKT and ERK1/2

**DOI:** 10.1371/journal.pone.0076836

**Published:** 2013-10-09

**Authors:** Rui Zeng, Yan Xiong, Fengming Zhu, Zufu Ma, Wenhui Liao, Yong He, JinSeng He, Wei Li, Juan Yang, Qian Lu, Gang Xu, Ying Yao

**Affiliations:** 1 Division of Nephrology, Tongji Hospital, Tongji Medical College, Huazhong University of Science and Technology, Wuhan, Hubei, China; 2 Division of Nephrology, Wuhan No.4 hospital, Wuhan, Hubei, China; 3 Department of Geriatrics, Tongji Hospital, Tongji Medical College, Huazhong University of Science and Technology, Wuhan, Hubei, China; 4 Division of Nephrology, Wuhan No.5 hospital, Wuhan, Hubei, China; National Center for Scientific Research Demokritos, Greece

## Abstract

Excess mesangial extracellular matrix (ECM) and mesangial cell proliferation is the major pathologic feature of diabetic nephropathy (DN). Fenofibrate, a PPARα agonist, has been shown to attenuate extracellular matrix formation in diabetic nephropathy. However, the mechanisms underlying this effect remain to be elucidated. In this study, the effect of fenofibrate on high-glucose induced cell proliferation and extracellular matrix exertion and its mechanisms were investigated in cultured rat mesangial cells by the methylthiazoletetrazolium (MTT) assay, flow cytometry and western blot. The results showed that treatment of mesangial cells (MCs) with fenofibrate repressed high-glucose induced up-regulation of extracellular matrix Collagen-IV, and inhibited entry of cell cycle into the S phase. This G1 arrest and ECM inhibition was caused by the reduction of phosphorylation and activation of extracellular signal-regulated kinase 1/2 (ERK1/2) and AKT. On the contrary, PPARα siRNA accelerated high glucose-induced cell cycle progression by ERK1/2 and AKT activation. Taken together, fenofibrate ameliorated glucose-induced mesangial cell proliferation and matrix production via its inhibition of PI3K/AKT and ERK1/2 signaling pathways. Such mechanisms may contribute to the favorable effects of treatment using fenofibrate in diabetic nephropathy.

## Introduction

Mesangial cell (MC) proliferation and excessive deposition of extracellular matrix proteins has been identified contributing to the progression of chronic kidney disease, including diabetic nephropathy (DN) [1]. Therefore, developing effective approaches to inhibit mesangial cell proliferation and extracellular matrix accumulation is of great importance for prevention of glomerular sclerosis in diabetes.

Fenofibrate is a peroxisome proliferator-activated receptor α (PPARα) agonist that has been widely used to treat dyslipidemia [2]. Interestingly, several studies revealed that fenofibrate ameliorated diabetes mellitus and diabetic microvascular damage beyond its lipid-lowering properties [3-6]. Fenofibrate has been identified to reduce levels of fibrinogen, C-reactive protein, and various pro-inflammatory markers, and improve flow-mediated dilatation [4], suggesting that fenofibrate exerts its clinical efficacy through PPARα-dependent and -independent mechanisms. Recent studies showed that fenofibrate plays a protective role in diabetic nephropathy [7,8]. It was identified to attenuate renal mesangial extracellular matrix formation [9], the pathological hallmark of diabetic nephropathy [10]. It was also demonstrated that fenofibrate significantly inhibited cell proliferation [11]. However, the underlying renoprotective mechanisms of fenofibrate in diabetic nephropathy have not been widely investigated.

There is growing evidence that mesangial cell proliferation and ECM accumulation play an important role in the pathogenesis of diabetic nephropathy, and that PI3K/AKT and ERK1/2 signaling pathways have been identified as key mediators of these events [11-14]. PI3-K/Akt and MAPKs pathways promote cell cycle progression by regulating cell cycle regulatory proteins cyclin-dependent kinase (CDK) 2 and CDK4 expression, which act in G1-S phase of cell cycle to induce cell proliferation in high glucose induced cell proliferation [15]. Fenofibrate was an ameliorator of PI3K/Akt signaling pathway [16,17]. Recently, Lee et al [11] had reported fenofibrate also inhibits mitogen-activated protein kinase (MAPK) signaling pathways such as ERK 1/2. Given the role of PPARα played in the regulation of PI3K/AKT and ERK/MAPK signaling, we therefore sought to more closely investigate the renoprotective effect of PPARa agonist, fenofibrate, in diabetic nephropathy by (1) determining its inhibition of glucose-induced mesangial cell proliferation and extracellular matrix accumulation; (2) examining its anti-proliferative and anti-ECM-secreting effects by an inhibitory effect of both the phosphorylation and activity of PI3K/AKT and ERK1/2 signaling pathways.

## Materials and Methods

### Reagents and Cell Culture

HBZY-1 cells (MCs), a rat mesangial cell line (purchased from Chinese Center for Typical Culture Collection, Wuhan, China), were cultured in DMEM supplemented with 10% fetal calf serum, 2mM glutamine, 100U/ml penicillin, and 100µg/ml streptomycin. After pre-incubation in DMEM supplemented with 0.1% fetal calf serum for 24 h, the cells were treated with indicated concentrations of glucose along with fenofibrate or MK886, a selective PPAR-α antagonist. The experiments were divided into following groups: normal glucose group (NG, 5mM glucose); high glucose group (HG, 40 mM glucose); high glucose and fenofibrate group (HG+FN, 40mM glucose and 100µM fenofibrate); high glucose and MK886 group (HG+ MK, 40mM glucose and 1µmol MK886). The cells were cultured for additional 12 h, 24 h or 48 h before harvesting. All cells were maintained in a 5% CO2 incubator at 37 °C. DMEM was purchased from GIBCO BRL (GIBCO, USA).

### PPAR-α Luciferase Reporter Assay

To measure activation of PPARα, a consensus PPAR response element (PPRE) fused to the upstream of the PGL2-basc luciferase reporter (p4xPPRE-Luc), a gift from molecular cancer research laboratory of University of Chicago, was used. MCs were seeded in a 24-well plate at a density of 5×10^4^ cells/well and incubated in DMEM low-sugar medium without antibiotics. The next day, the cells were transfected with 100ng of pRL-TK vector and 500ng of p4xPPRE-Luc using lipofectamine 2000 (Invitrogen) and OPTI-MEM (Invitrogen). Five hours later, the cells were treated with indicated concentration of fenofibrate or MK886 for 24 h, followed by analysis of Firefly luciferase and Renilla luciferase activities using a dual-luciferase reporter assay kit (Promega, USA).

### Cell proliferation assay

The MTT assay was used as a qualitative index of cell proliferation. We cultured MCs cells in 96-well plates (5 × 10^3^ cells/well). After 12 h, 24 h or 48 h incubation with above indicated compounds, 20µl MTT (5 mg/ml, Invitrogen, USA) was added and the cells were cultured for additional 4h. Subsequently, the cells were lysed using dimethylsulfoxide (150μl/well, Sigma, USA). When the formanzan crystals were completely dissolved, the optical density (OD) was measured at 570 nm using a Microplate Reader (Biotek synergy, USA).

### Flow cytometry

Cell cycle analysis was performed by flow cytometry. The cells were harvested 24h after treatment and fixed with −20°C 70% ethanol. After washes the cells were treated with RNase (25 µg/ml) at 37 °C for 30 min, followed by staining with propidium iodide (PI, 50µg/ml) at 4°C for 30 min in the dark. The cells were analyzed using a FACS Calibur Flow Cytometer (BD, USA) to determine the proportion of cells within the G_1_, S, and G_2_/M phases.

### RT-PCR

Cell total RNA was extracted with TRIZOL reagent (Invitrogen) according to the manufacturer’s directions. One microgram of total RNA was reverse transcripted into cDNA according to the manufacture’s instruction (Promega, USA). Primers for rat PPARα are 5' GCCGTTTCCACAAGTGCC 3' and 5' TGCTAGTCTTTCCTGCGAGTATG 3', and primers for rat CDK-4 are 5' CCCAATGTTGTACGGCTGAT 3' and 5' GAGGTGCTTTGTCCAGGTATGT 3', and primers for rat Beta-actin are 5' TCTTCCAGCCTTCCTTCCTG 3' and 5' AGAGCCACCAATCCACACA 3'. A two-step PCR was performed for 35 cycles. Denaturation was performed at 95°C for 10 s, annealing/extension at 55°C for 30 s. Measurements were standardized to the expression of the beta-actin. Relative gene expression is reported after normalizing to that cultured in low glocuse.

### ELISA assay

Collagen-IV secretions were measured using an ELISA kit (R&D Systems) according to the manufacturer’s instructions. Each sample was analyzed in triplicates, and the absorbance was measured at 450 nm wavelength and calculated in the linear.

### Western blotting

The cells were lysed with RIPA lysis buffer containing PMSF protease inhibitors. Protein concentrations were determined using the BCA method (Beyotime Institute of Biotechnology, China). The proteins were first separated by SDS-PAGE, and transferred onto nitrocellulose membranes. After blocked with 5% non-fat milk in Tris-buffered saline-Tween20 (TBS-T, pH7.6) at room temperature, the membranes were incubated overnight at 4 °C with indicated primary antibodies (PPARα,1:500,; GAPDH,1:1000; Collagen-IV,1:50;phosphor-ERK1/2, 1:500; ERK1/2 1:500; phosphor-AKT,1:200; and AKT,1:500). After washing, the membranes were incubated with corresponding secondary antibodies and then developed using the ECL western blotting reagents. The relative intensity of each target band was quantified by the BioSpectrum UVP imaging system, and normalized by the intensity of GAPDH. ERK1/2 and AKT activation was assessed by the ratio of phosphorylated form among its corresponding total protein levels.

#### SiRNA

HBZY-1 cells were transfected with 100nM PPARα-specific siRNA (Invitrogen, 5’-3’ GAGAUCGGCCUGGCCUUCUAAACAU) using Lipofectamine 2000 (Invitrogen) according to the manufacturer’s instructions. Briefly, HBZY-1 cells (1×105/mL) were seeded in 6-well plates with siRNA-Lipofectamine complexes. 5 h after transfection, cells were treated with high glucose and/or LY294002 (10 µmol/L), or U0126 (10 µmol/L) for another 24 hours. Total RNA and protein were extracted from the cells for analysis of PPARα, Col-IV, p-Akt and pERK1/2 expression. A nonsilencing siRNA oligonucleotide that does not recognize any known homology to mammalian genes (Invitrogen) was used as a negative control. All experiments were repeated three times.

### Statistical analysis

All data are expressed as mean ± SD and were compared by one-way ANOVA with post-hoc Bonferroni’s correction (GraphPad Prism 5.0; GraphPad Software). In all cases, *p* < 0.05 was considered statistical significance.

## Results

### PPARα was upregulated and activited in rat mesangial cells treated with high glucose and/or fenofibrate

**Figure 1:**
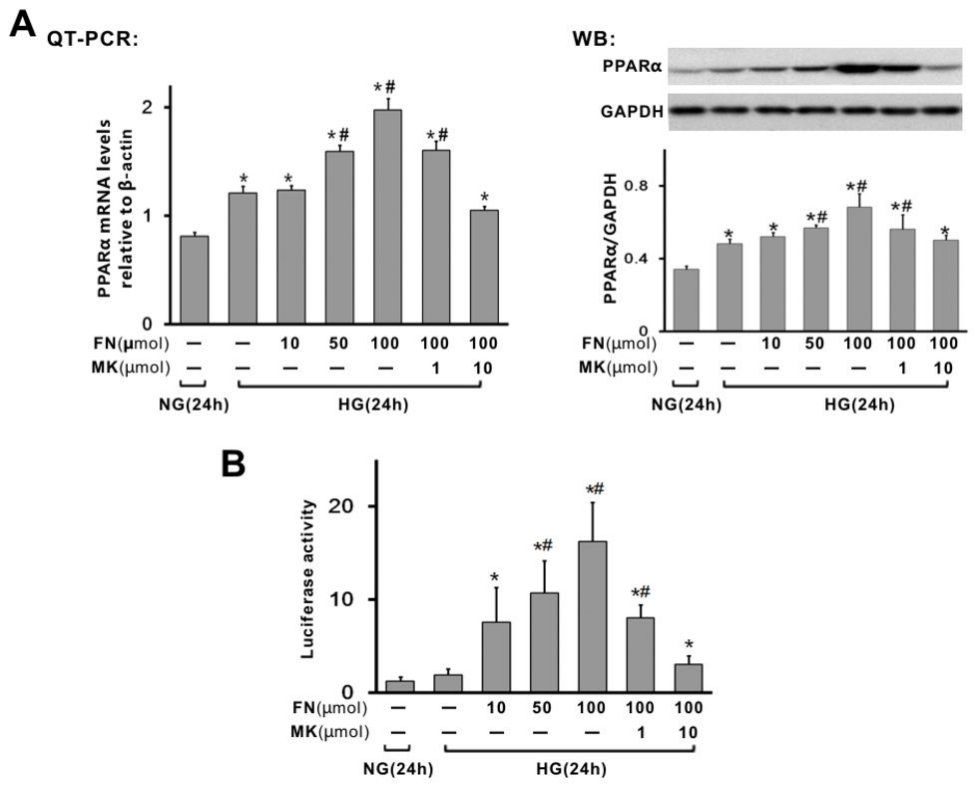
Expression and activity of PPARα in rat mesangial cells (MCs) treated with high glucose and/or fenofibrate. Mesangial cells were stimulated with high glucose 40mmol/L, in the absence or presence of fenofibrate (10–100 µM), or 100 µM fenofibrate plus MK886 (1 or 10 µM) for 24 h. (A) After incubation, PPARα mRNA and protein levels were determined by real time RT-PCR and Western Blot, normalized to β-actin or GAPDH. (B) For detection of PPARα activity, MCs were transfected with a PPRE-luciferase reporter vector for 5 h before treatment. Its activity was measured using the luciferase assay system. Data in the bar graphs represent the average values ± SE of experiments performed in triplicate. *P<0.05 vs NG; ^#^ p<0.05 vs HG. NG, normal glucose (5mM); HG, high glucose (40 mM); FN, fenofibrate; MK, MK886.

To explore the role of fenofibrate on high glucose induced mesangial cell proliferation and extracellular matrix accumulation, we first assessed the presence of PPARα in high glucose treated MCs by mRNA and Western blot. As is shown in Figure 1A, PPARα messenger RNA and protein was detectable in non-stimulated cells. High glucose stimulation (40mmol/L for 24h) induced a slight increase of PPARα mRNA and protein in MCs. However, treatment with fenofibrate resulted in a significant dose-dependent induction of PPARα expression in high glucose treated MCs, with a maximum of induction after 24 h with 100 µM (Figure 1A). The enhancing effect was reversed by the administration of 1 or 10 µmol/L MK886 (Figure 1A), a selective PPAR-α antagonist.

In addition, we estimated the activity of PPARα by transiently transfecting MCs with a PPRE-luciferase reporter construct (Figure 1B). MCs were transfected with the p4XPPRE-Luc expression vector, treated with high glucose in the absence or presence of fenofibrate (1, 10, 100µM) or MK886 for 24 h, and then analysed for luciferase activity as PPRE-mediated transcriptional activity, specific PPARα activity. As shown in Figure 1B, treatment with fenofibrate enhanced PPRE-mediated transcriptional activity compared to that without fenofibrate treatment. This enhancing activity was inhibited by the administration of MK886 (Figure 1B). Those data suggested the presence of a functioning PPARα protein and its actual activation by fenofibrate in high glucose treated MCs.

### Fenofibrate Suppressed HG-Induced Rat Mesangial Cell Proliferation

**Figure 2:**
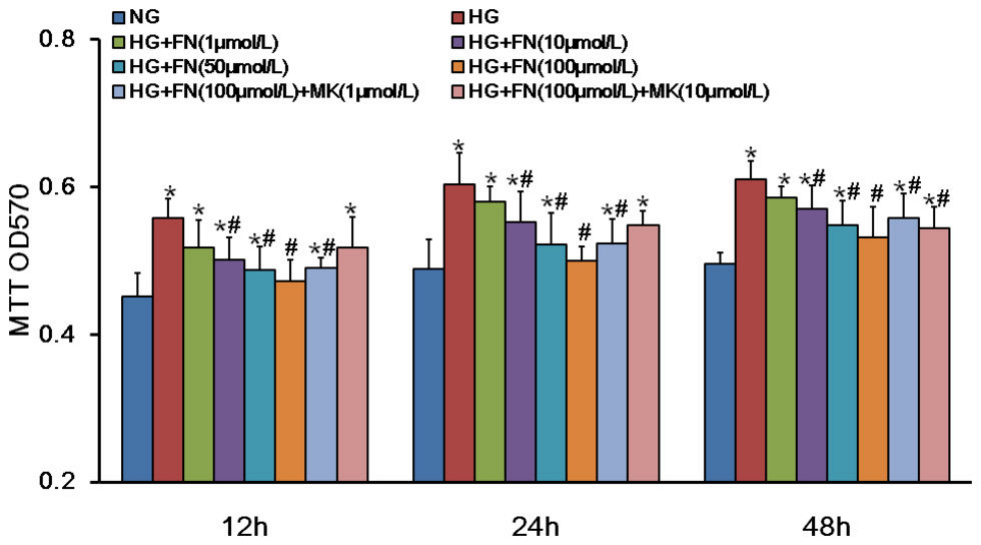
MTT assay for cell proliferation analysis. Fenofibrate inhibited mesangial cell proliferation induced by high glucose for 12h, 24 h or 48h, at a final concentration 1, 10, 50, 100µM, respectively. The maximum inhibition was reversed by the administration of MK886 (1 or 10 µmol/L). The result is average values ± SE of representative data from experiments in triplicate. ^*^P<0.05 vs NG; ^#^ p<0.05 vs HG. NG, normal glucose (5mM); HG, high glucose (40 mM); FN, fenofibrate; MK, MK886.

MTT assay was employed to assess the impact of Fenofibrate on MC proliferation. It was interestingly noted that administration of Fenofibrate, no matter for 12h, 24 h or 48h, significantly suppressed high glucose induced MC proliferation, with a maximum of inhibition in 100 µmol/L (Figure 2). The inhibition was inhibited by the administration of MK886 (1 or 10 µmol/L).

### Fenofibrate arrested HG-induced cell-cycle progression in rat mesangial cells

**Figure 3:**
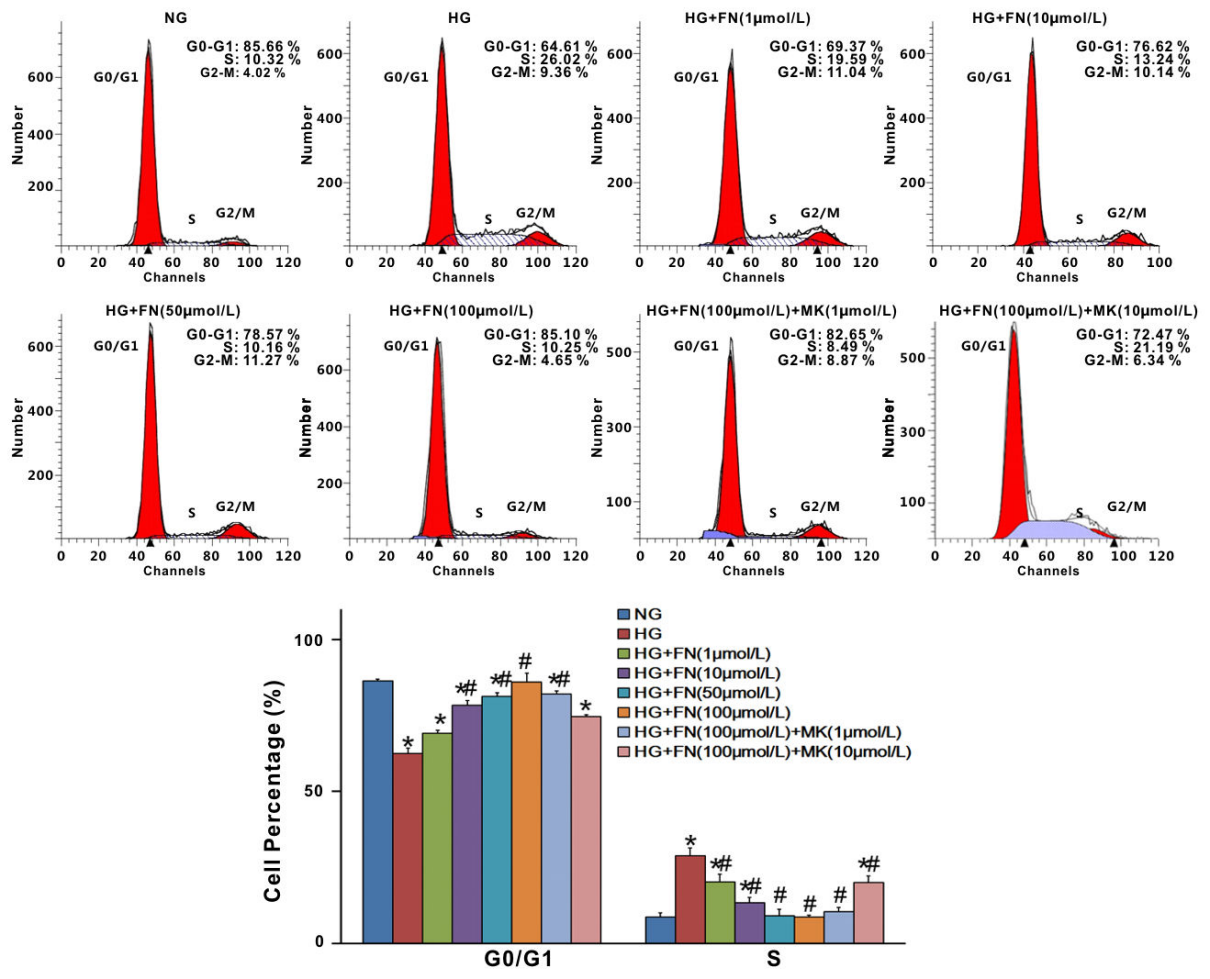
Fenofibrate reduced mesangial cell proliferations stimulated by high glucose. MCs were preincubated for 2h with various concentrations of fenofibrate, and then stimulated with high glucose for 24h. Fenofibrate inhibits mesangial cell proliferation induced by high glucose at a final concentration 1, 10, 50, 100µM, respectively. Cells were analyzed by flow cytometry after PI staining and the relative percentage of cells in different cell cycle phases are reported, while the percentage of apoptotic events was ignored. Data in the bar graphs represent the average values ± SE of experiments performed in triplicate. ^*^P<0.05 vs NG; ^#^ p<0.05 vs HG. NG, normal glucose (5mM); HG, high glucose (40 mM); FN, fenofibrate; MK, MK886.

To further examine the role of fenofibrate in MC cell cycle progression, we did flow cytometry analysis. As shown in Figure 3, high-glucose induced decrease of cells in G1 phase, but increased cells in S phase, indicating that high glucose promotes cell cycle progression. In contrast, fenofibrate dose-dependently arrested cells in G1 phase (p<0.05), and therefore, the number of cells in S phase was significantly lower (p<0.05). This fenofibrate-induced cell growth arrest was reversed by MK886 (Figure 3). These results suggest that fenofibrate could block high glucose induced cell cycle progression by inhibiting the G1-S phase transition and arresting cells in G1 phase.

### Fenofibrate suppressed high glucose induced expression of Collagen-IV in rat mesangial cells

**Figure 4:**
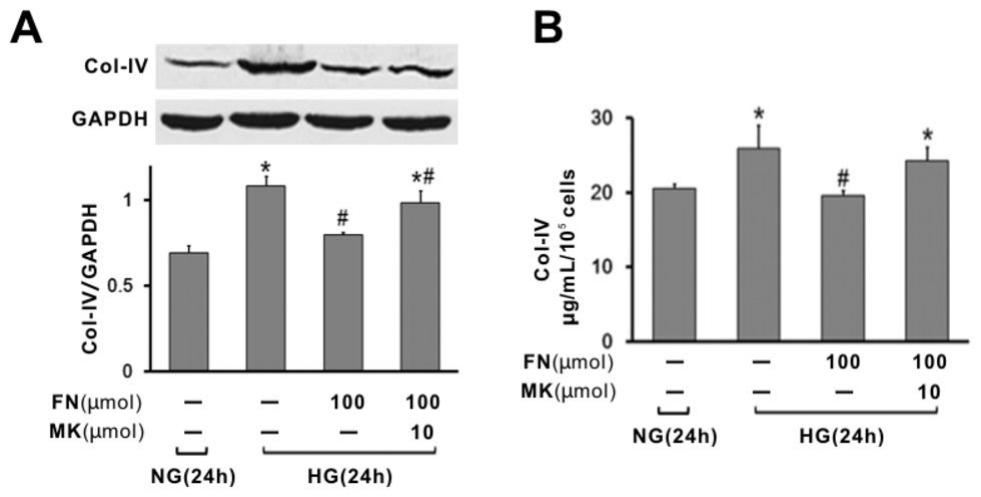
Effect of fenofibrate on the expression and secretions of extracellular matrix component in high glucose treated mesangial cells. (A) Representative western blot of Collagen-IV. (B) Collagen-IV secretion in culture media by ELISA assay. Protein levels were determined by Western Blot, normalised to GAPDH. Values are given as mean ±S.D. from 3 independent experiments in triplicate and p<0.05 is considered statistically significant. ^*^P<0.05 vs NG; ^#^ p<0.05 vs HG. NG, normal glucose (5mM); HG, high glucose (40 mM); FN, fenofibrate; MK, MK886.

As matrix accumulation was a consequence of mesangial cells proliferation, matrix components expressions and secretions in MC cells were measured. Western blot analysis revealed that high glucose induced high levels of Collagen-IV expression in MCs as compared with that of control cells (Figure 4A). Administration of fenofibrate significantly attenuated the expression of Collagen-IV induced by high glucose. Collagen-IV secretions were detected by ELISA assay in culture supernatants. In consistent with the above results, the levels of Collagen-IV in HG-treated cells were significantly higher than that of control cells (Figure 4B), but attenuated by fenofibrate treatment (*P* < 0.05). The inhibition of matrix component expression and secretion by fenofibrate was reversed by the administration of MK886 (Figure 4). Those data suggested fenofibrate suppressed high glucose induced matrix accumulation in rat mesangial cells through the activation of PPARα protein.

### Fenofibrate suppressed ERK1/2 and AKT activation in rat mesangial cells

**Figure 5:**
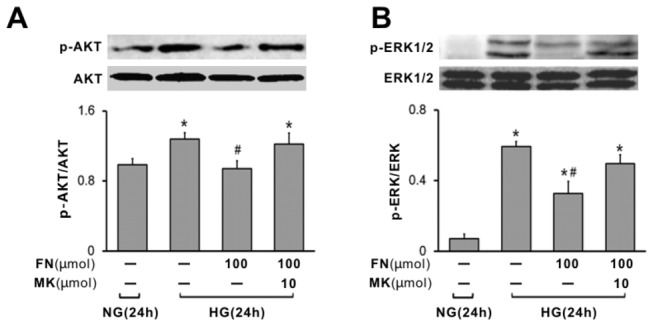
Effect of fenofibrate on the phosphorylation of ERK1/2 and PI3K-AKT in rat mesangial cells. (A) Representative western blot of p-AKT. (B) Representative western blot of p-ERK1/2. The figure shows the average volume density of phosphorylated ERK1/2 and AKT corrected for the loading control, total ERK1/2 and AKT. Values are given as mean±S.D. from 3 separate experiments. ^*^P<0.05 vs NG; ^#^ p<0.05 vs HG. NG, normal glucose (5mM); HG, high glucose (40 mM); FN, fenofibrate; MK, MK886.

Given the role of ERK1/2 and AKT signaling played in mesangial cell growth and proliferation, we examined the impact of fenofibrate on ERK1/2 and AKT activation. It was noted that high glucose induced ERK1/2 and AKT activation as manifested by that the relative amount of phosphorylated ERK1/2 and AKT is significantly higher than that of control cells (Figure 5). However, treatment of fenofibrate significantly suppressed high glucose induced ERK1/2 and AKT activation. This inhibition was reversed by MK886.

**Figure 6:**
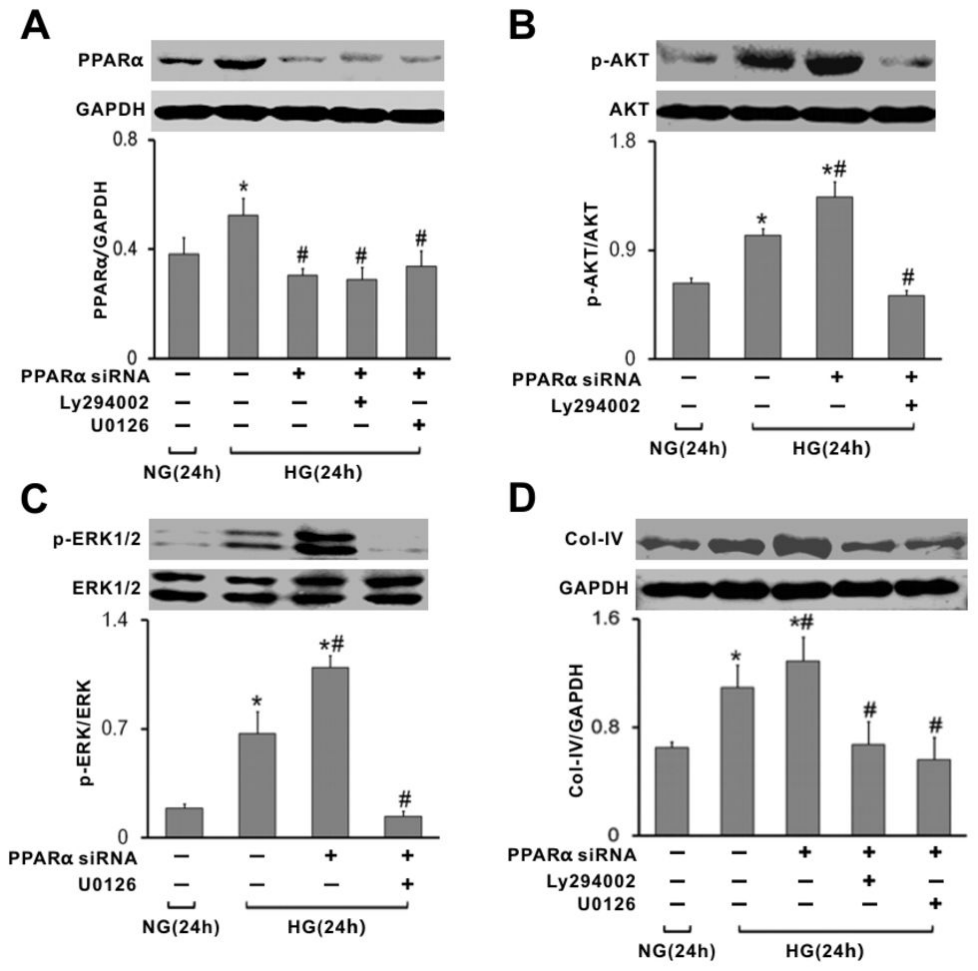
Effect of PPARα siRNA on ERK1/2 and PI3K-AKT pathways, and the Collagen-IV expression in high glucose treated mesangial cells. (A) Representative western blot of PPARα. (B) Representative western blot of p-AKT. (C) Representative western blot of p-ERK1/2. (D) Representative western blot of Collagen-IV. Protein levels were determined by western Blot, normalised to GAPDH. Phosphorylated ERK1/2 and AKT were corrected for the loading control, total ERK1/2 and AKT. Values are given as mean ±S.D. from 3 independent experiments in triplicate and p<0.05 is considered statistically significant. ^*^P<0.05 vs NG; ^#^ p<0.05 vs HG. NG, normal glucose; HG, high glucose.

We further investigated the effect of PPARα siRNA on high glucose-induced ERK1/2 and PI3K-AKT activation, and Collagen-IV expression. Treatment with PPARα siRNA significantly inhibited PPARα expression (Figure 6A), and resulted in an increased phosphorylation of AKT (Figure 6B) and ERK1/2 (Figure 6C) in high glucose treated mesangial cells. These phosphorylations were reversed by AKT inhibitor, LY294002, and ERK inhibitor, U0126 (Figure 6B and 6C). PPARα siRNA further upregulated the expression of Collagen-IV, compared to that of high glucose treated cells (Figure 6D). Administration of either AKT inhibitor or ERK inhibitor attenuated the expression of Collagen-IV induced by high glucose and PPARα knockdown (Figure 6D). Those data suggested PPARα siRNA accelerated high glucose-induced ERK1/2 and PI3K-AKT activation in rat mesangial cells.

**Figure 7:**
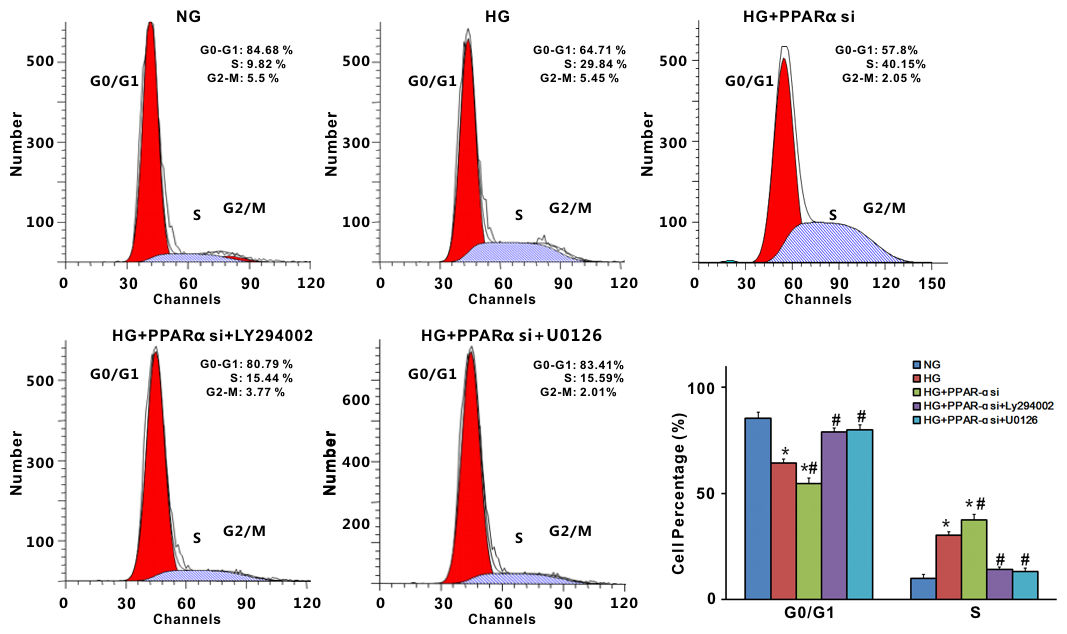
PPARα siRNA accelerated high glucose-induced mesangial cell proliferations via ERK1/2 and PI3K-AKT pathways. PPARα siRNA was transfected into mesangial cells for 5h, followed by stimulating with high glucose for 24h, or high glucose plus Ly294002 (10µmol/L), or high glucose plus U0126 (10µmol/L). Cells were analyzed by flow cytometry after PI staining. Data in the bar graphs represent the average values ± SE of experiments performed in triplicate. ^*^P<0.05 vs NG; ^#^ p<0.05 vs HG. NG, normal glucose; HG, high glucose.

We further examined cell cycle progression in these cells. As shown in Figure 7, PPARα siRNA promoted G1 phase cell cycle progression during high glucose treatment, and the number of cells in S phase increased (p<0.05). This PPARα siRNA-induced cell growth was inhibited by treatment of either AKT inhibitor or ERK inhibitor (Figure 7).

**Figure 8:**
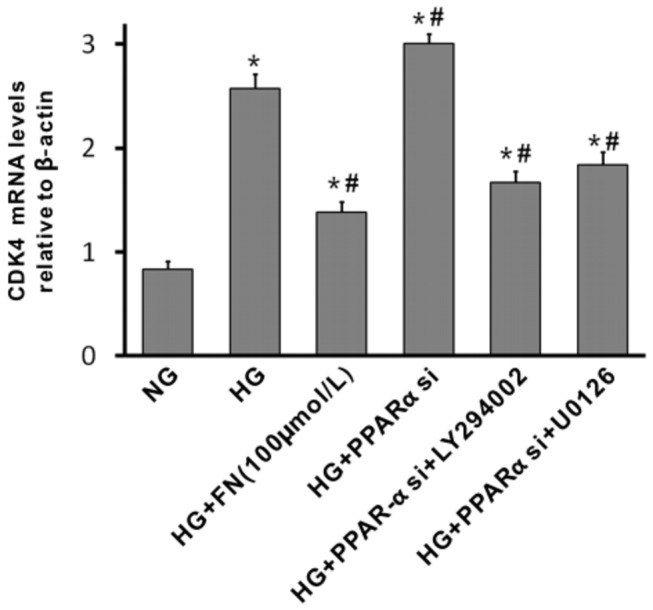
Expression CDK4 in rat mesangial cells (MCs) treated with high glucose. Mesangial cells were stimulated with high glucose 40mmol/L, in the absence or presence of fenofibrate (100 µM), or PPARα siRNA plus Ly294002 (10µmol/L), or high glucose plus U0126 (10µmol/L) for 24 h. CDK4 mRNA levels were determined by real time RT-PCR, normalised to β-actin. Data in the bar graphs represent the average values ± SE of experiments performed in triplicate. *P<0.05 vs NG; ^#^ p<0.05 vs HG. NG, normal glucose; HG, high glucose; FN, fenofibrate.

In view of the fact that PPARα directly or indirectly affects transcription of genes such as CDK-4, which is essential for cell cycle progression, we investigated the CDK-4 expression in these cells by real time RT-PCR. As is shown in Figure 8, high glucose stimulation induced an increase of CDK-4 mRNA in MCs. Treatment with fenofibrate inhibited high glucose-induced CDK-4 upregulation (Figure 8), which was accelerated by PPARα siRNA. Administration of AKT inhibitor or ERK inhibitor attenuated the expression of CDK-4 induced by high glucose and/or PPARα knockdown (Figure 8). It suggested that CDK4 inhibition is one of the mechanisms of PPARα regulated mesangial cell proliferation.

As PI3K/AKT and ERK1/2 signaling pathways have been identified as key mediators of CDK-4 expression, cell proliferation and ECM accumulation, these results suggest that PPARα regulates glucose-induced mesangial cell proliferation and matrix production via PI3K/AKT and ERK1/2 pathways.

## Discussion

Although fenofibrate has been identified to have renal protective effects in diabetic nephropathy [18], it is not clear which role fenofibrate plays in glucose induced mesangial cells (MCs) proliferation, a characteristic for early stage of diabetic nephropathy [19]. In this study, we identified the inhibitory effect of fenofibrate on high glucose induced rat MCs proliferation. We found treatment of mesangial cells (MCs) with fenofibrate repressed high-glucose induced up-regulation of extracellular matrix Collagen-IV, and inhibited entry of cell cycle into the S phase.

Fenofibrate arrested cell cycle of high glucose treated MCs from G1 to S phase transition in a time and dose-dependent manner. During cell cycle progression, S-phase is synthesis phase in which DNA is replicated, after which, the cells enter into G2 phase, and finally mitosis occurs in M phase. Compared with cells cultured under normal concentration of glucose, cells under high-glucose condition showed significantly higher population at S phase, but lower proportion of cells at G1-phase. Treatment of fenofibrate (100µM for 24h), almost completely blocked the high glucose induced G1 to S phase progression of the cell cycle.

Extracellular matrix production (ECM) is a key event in the progression of renal failure [20]. When exposed to a variety of high glucose, mesangial cells synthesize extracellular matrix proteins, which lead to the development of progressive renal disease and finally glomerulosclerosis in humans and animal diabetic models [21]. Our results demonstrated that high glucose enhanced MCs proliferation accomplishing an increase of ECM production rate. Treatment of fenofibrate time- and dose-dependently ameliorated HG-induced cell proliferation and expression of Collagen-IV protein, suggesting that fenofibrate is an effective agent for preventing the development of glomerulus sclerosis in diabetes.

Despite extensive efforts in research during recent years, the molecular mechanisms underlying anti-proliferation and anti-ECM production effects of fenofibrate in mesangial cells remain poorly understood. Our obsevations in this study were reflecting the specific action of fenofibrate on cell cycle progression rather than induction of apoptosis, implying that the antiproliferative properties of fenofibrate were caused by the modulation of signaling pathway involved in cell cycle progression.

The serine/threonine kinase Akt and extracellular signal-regulated kinase (ERK) 1/2 are the two major signaling molecules in the regulation of ECM protein synthesis and cell proliferation [22,23]. It has been reported that the PI3K-AKT and ERK1/2 signaling pathways were activated in glomeruli of diabetic rats as well as in mesangial cells cultured under high-glucose conditions [12,24]. These observations prompted us to examine the impact of fenofibrate on PI3K-AKT and ERK1/2 MAP kinase signaling. We found that high glucose enhanced the levels of phosphorylated AKT and ERK1/2 in mesangial cells. Fenofibrate treatment significantly decreased the levels of p-ERK and p-AKT in high-glucose treated MCs. Recently, Lee JJ et al demonstrated fenofibrate suppressed the G0/G1 to S phase of cell cycle in neointimal formation through ERK 1/2 signaling pathway, which resulted in the downregulation of cyclin D, cyclin E, CDK2, CDK4, PCNA proteins, and finally induced the G0/G arrest [11]. However, they hadn’t observed the PI3K-Akt signaling cascade, another important G1 phase arrest signaling pathway [25], was involved. Our results indicate that fenofibrate not only inhibited the activation of MAPK signaling pathways, but also suppressed the phosphorylation of Akt protein in high glucose treated MCs. Through knockdown of PPARα by siRNA, we found PPARα inhibition accelerated high glucose-induced mesangial cell proliferation and phosphorylation of ERK and AKT, and was accomplished with upregulated CDK4 expression, compared to the inhibitory effect of fenofibrate. All these were attenuated by either AKT inhibitor or ERK inhibitor, suggesting PPARα regulates glucose-induced mesangial cell proliferation via both PI3K/AKT and ERK1/2 pathways. Thus, fenofibrate, as a PPARα agonist, may be sufficient to achieve cell cycle arrest in the inhibitory action of high glucose-mediated proliferation of MCs.

In summary, our data provided novel insight into the understanding of the molecular and signaling pathway mechanism, in which fenofibrate suppresses high glucose induced activation of both ERK1/2 and AKT, and by which it arrests MCs at G1 phase. Thus, fenofibrate is a potential therapeutics for prevention and treatment of early stage diabetic nephropathy in the clinical setting.
